# Contributions to Loss Across the Magnetopause During an Electron Dropout Event

**DOI:** 10.1029/2022JA030751

**Published:** 2022-10-08

**Authors:** H. George, G. Reeves, G. Cunningham, M. M. H. Kalliokoski, E. Kilpua, A. Osmane, M. G. Henderson, S. K. Morley, S. Hoilijoki, M. Palmroth

**Affiliations:** ^1^ Department of Physics University of Helsinki Helsinki Finland; ^2^ Intelligence and Space Research Division Los Alamos National Laboratory Los Alamos NM USA; ^3^ Space and Earth Observation Center Finnish Meteorological Institute Helsinki Finland

**Keywords:** radiation belts, dropout, radial diffusion, drift orbit bifurcation

## Abstract

Dropout events are dramatic decreases in radiation belt electron populations that can occur in as little as 30 minutes. Loss to magnetopause due to a combination of magnetopause shadowing and outward radial transport plays a significant role in these events. We examine the dropout of relativistic electron populations during the October 2012 geomagnetic storm using simulated electron phase space density, evaluating the contribution of different processes to losses across the magnetopause. We compare loss contribution from outward transport calculated using a standard empirical radial diffusion model that assumes a dipolar geomagnetic field to an event‐specific radial diffusion model evaluated with a non‐dipolar geomagnetic field. We additionally evaluate the contribution of Shabansky type 1 particles, which bounce along magnetic field lines with local equatorial maxima, to the loss calculated during this event. We find that the empirical radial diffusion model with a dipolar background field underestimates the contribution of radial diffusion to this dropout event by up to 10% when compared to the event‐specific, non‐dipolar radial diffusion model. We additionally find that including Shabansky type 1 particles in the initial electron phase space density, that is, allowing some magnetic field lines distorted from the typical single‐minima configuration in drift shell construction, increases the calculated loss by an average of 0.75%. This shows that the treatment of the geomagnetic field significantly impacts the calculation of electron losses to the magnetopause during dropout events, with the non‐dipolar treatment of radial diffusion being essential to accurately quantify the loss of outer radiation belt populations.

## Key Points

- Geomagnetic field treatment greatly affects loss quantification during dropout events
- Non‐dipolar radial diffusion models are required to precisely quantify loss across the magnetopause
- Shabansky particles may significantly contribute to total loss across the magnetopause

## 1 Introduction

The outer radiation belt can be dramatically altered by dropout events, which deplete electron fluxes by up to several orders of magnitudes in a few hours or even less (Morley et al., 2010; Olifer et al., 2018; Ozeke et al., 2017; Turner, Morley, et al., 2012). Dropout events typically occur during geomagnetic storms and represent the permanent (non‐adiabatic) loss of electrons from the radiation belts (Shprits et al., 2006), which occurs either via electron precipitation to the upper atmosphere or by loss across the magnetopause to the solar wind (Turner, Shprits, & Angelopoulos, 2012). In the latter mechanism, the drift orbits of previously trapped particles intersect the magnetopause due to the inward motion of the magnetopause (magnetopause shadowing) and/or radially outward motion of the electrons (Loto'aniu et al., 2010; Shprits et al., 2006; Turner et al., 2012; Xiang et al., 2017). Dropout events can effectively reset the outer radiation belt environment, particularly for relativistic electrons that can almost completely disappear.

The magnetopause location is one of the key factors determining the amount of loss to the solar wind, with the greater loss occurring when the magnetopause is compressed (e.g., Kim et al., 2008; Yu et al., 2013). Strong solar wind forcing during geomagnetic storms typically leads to effective magnetopause shadowing, with the greatest magnetopause compression and erosion occurring during periods of southward interplanetary magnetic field (IMF) and elevated solar wind dynamic pressure (Shue et al., 1998). In extreme cases, the dayside magnetopause can be compressed within the geosynchronous orbit (e.g., Dmitriev et al., 2005). Subsequently, the strongest dropouts of relativistic electrons during geomagnetic storms are statistically associated with both elevated dynamic pressure and southward IMF (Boynton et al., 2016; Yuan & Zong, 2013). Loss to the magnetopause is a major contributor to loss of relativistic electrons at mid‐ and high L‐shell (e.g., at geostationary orbit, Boynton et al., 2016) although precipitation into the upper atmosphere is also an important contributor to dropout events, particularly at lower L‐shell (*L* ≤ ∼ 4.2, Boynton et al. [2017]; Xiang et al. [2018]). The precipitation of relativistic electrons is likely predominantly driven by chorus and/or electromagnetic ion cyclotron waves (Drozdov et al., 2022; Mourenas et al., 2016). This study focuses on the outward losses to the magnetopause during a dropout event, that is, without considering loss via precipitation, and how the treatment of the geomagnetic field affects the evaluation of this outward loss.

The dynamics of a given radiation belt population are often clearer when expressed as phase space density (PSD) rather than flux. PSD is a function of the three adiabatic invariants, *μ*, *K*, and L*, which correspond to the Larmor, bounce, and drift motion of a particle respectively. PSD allows for the isolation of a single radiation belt electron population (Hartley & Denton, 2014), where a population is defined here as a subset of electrons with the same adiabatic invariants. The spatial and temporal evolution of the PSD profile can thus be used to identify transport, enhancement, and loss processes acting on a specific population (e.g., Reeves et al., 2013). This is particularly useful when evaluating loss to the magnetopause, as particles with different *μ* and *K* values that are initially on the same field line in a non‐dipolar field will experience drift shell splitting and therefore have different L* and different trajectories in space. Consequentially, the intersection of drift orbits with the magnetopause is dependent on the adiabatic invariants of the population, despite the magnetopause being at a given position at a given time. PSD also enables the distinction between adiabatic and non‐adiabatic processes, or, put differently, the distinction between reversible and irreversible transport processes. The effect of adiabatic transport processes, such as the Dst effect, on a population, can be misleading when evaluating dropout events as they can result in a reversible decrease in fluxes of a given energy. The PSD remains constant during adiabatic processes due to the conserved adiabatic invariants, so examination of PSD rather than flux reveals the irreversible losses that occur during dropouts.

The last closed drift shell (LCDS) is the largest L* for a population with a given *K* value that does not intersect the magnetopause (Albert et al., 2018; Roederer & Zhang, 2014). The LCDS, therefore, provides a population‐specific magnetopause proxy in adiabatic space, allowing for direct comparison with the PSD. Populations at L* less than or equal to the LCDS are trapped within the radiation belt, while more distant populations have drift paths that intersect the magnetopause and so are lost from the radiation belts. The use of the LCDS allows for a more accurate and efficient evaluation of the loss of a specific population to the solar wind than the spatial magnetopause location (e.g., Olifer et al., 2018; Tu et al., 2019; Xiang et al., 2017) and provides a reliable indicator of the intensity and depth of a dropout event (Olifer et al., 2021). Therefore, the use of PSD in combination with LCDS can be used to more rigorously determine the loss to the magnetopause experienced by a single electron population.

Radial diffusion is a non‐adiabatic transport process during which particles are diffused across drift shells due to the violation of the third adiabatic invariant (equivalently, violation of L*). Populations undergoing radial diffusion are diffused from regions of high PSD to regions of low PSD, so if there is a peak of PSD at a location inside the radiation belts, then there will be net outward transport toward the magnetopause at locations beyond that peak. We note that radial transport can also redistribute and enhance populations that remain trapped during geomagnetic storms (e.g., Reeves et al., 1998). Radial diffusion is predominantly driven by waves in the ultralow frequency (ULF) Pc5 range (periods of 150–600 s, Jacobs et al. [1964]), as these waves can drift‐resonate with energetic outer radiation belt electrons (Elkington et al., 1999) and thus are well suited to violate L*. Magnetospheric ULF Pc5 wave occurrence is strongly linked to periods of high solar wind speed (Mathie & Mann, 2001) and dynamic pressure (Kessel, 2008), and radial diffusion is in turn enhanced during periods of elevated driving wave activity. Therefore, during periods of strong solar wind forcing, losses to the magnetopause are expected to be particularly strong due to the combined effect of inward magnetopause motion and enhanced outward radial transport.

Radial diffusion coefficients are used to quantify the amount of transport due to radial diffusion. There are a number of models that calculate the radial diffusion coefficients (e.g., Ali et al., 2016; Ozeke et al., 2014; Selesnick et al., 1997) and the choice of radial diffusion model can have significant impact. Tu et al. (2019), for example, simulated a dropout event with two different radial diffusion models and found that one radial diffusion model lead to simulated PSD that was up to an order of magnitude greater than in‐situ observations immediately after the dropout, while the other radial diffusion model produced simulated PSD that was much more similar to the observations. Many radial diffusion models evaluate radial transport over the L‐shell parameter, which is equivalent to equatorial radial distance in Earth radii, rather than L*. L‐shell and L* are numerically equal in dipolar geomagnetic fields, but they diverge as the field becomes increasingly distorted from the dipolar configuration. Here we refer to radial diffusion coefficients using L‐shell (numerically equivalent to using L* in a dipole background field) as *D*_*LL*_ and radial diffusion coefficients using L* in a non‐dipolar background field as *D*_L*L*_. Significantly different radial diffusion coefficients can be obtained from the same ULF wave model during the same event when evaluated with dipolar or non‐dipolar geomagnetic fields (Cunningham, 2016). As the geomagnetic field is significantly non‐dipolar at both high radial distances and during strong solar wind forcing, particles that are lost to the magnetopause during dropout events would sample highly non‐dipolar regions of the Earth's magnetic field. One of the objectives of this study is to evaluate the treatment of the Earth's magnetic field as either dipolar or non‐dipolar during a dropout event to determine the impact that this treatment has on the calculated loss of outer radiation belt electrons. The need for evaluation of loss during dropout events with event‐specific radial diffusion coefficients has previously been identified in Tu et al. (2019), which we also address in this study.

Drift orbit bifurcation refers to the splitting of electron drift shells due to magnetic field asymmetries on the dayside of the Earth (Shabansky, 1971). Magnetic field lines can form two local minima on either side of the equatorial plane of the Earth, which typically occurs within 1 – 2*R*_*E*_ of the dayside magnetopause (Öztürk & Wolf, 2007), while those on the nightside maintain their typical (single minima) configuration (Mead & Beard, 1964). Particles that bounce along these magnetic field lines with local maxima can be divided into Shabansky type 1, 2, or 3 orbits depending on their characteristic trajectories, following the naming system introduced in McCollough et al. (2012). Shabansky type 1 particles travel along magnetic field lines with local maxima at the equator that remains below the magnetic field mirror value for that population. This means that Shabansky type 1 particles still bounce between the magnetic mirror points close to the ionosphere at both the northern and southern hemispheres (McCollough et al., 2012). Shabansky type 1 particles have conserved *K* and L* values due to their complete bounce motion. Shabansky type 2 and 3 particles execute the bifurcated drift orbits described in Shabansky (1971), where they become confined to a single hemisphere and only bounce between the local maxima and a magnetic mirror point near the ionosphere in either the northern or southern hemisphere. Both Shabansky type 2 and 3 particles violate the second adiabatic invariant upon drift orbit bifurcation (Antonova et al., 2003), with the key difference between the two orbit types arising from the evolution of the second adiabatic invariant in the region of bifurcated magnetic fields (McCollough et al., 2012). The breaking of the particles' bounce motion additionally means that L* is no longer defined for Shabansky type 2 or 3 particles. We note that it is possible to compute L* for bifurcated orbits by estimating the division of *K* between the two branches but this technique is still experimental and requires strong approximations on the partitioning of *K*. Drift orbit bifurcation can result in transport in the absence of driving wave activity, and this transport can become competitive with radial diffusion of ultrarelativistic particles at high radial distances (Öztürk & Wolf, 2007). The violation of *K* leads to both ballistic and diffusive radial transport (Ukhorskiy et al., 2011), which can increase the radial transport rates by up to an order of magnitude from the scenario with only ULF‐driven radial diffusion (Ukhorskiy et al., 2014). The long‐term (timescales corresponding to many drift orbits) radial transport resulting from drift orbit bifurcation is strongly driven by the convective electric field and can directly lead to permanent loss through magnetopause (Desai et al., 2021). Therefore, it is possible that a significant portion of electrons lost to the magnetopause during dropout events have Shabansky type orbits, both due to the locations where drift orbit bifurcation typically occurs and due to the resulting radial transport. The challenges associated with modeling these complex orbits, however, mean that electrons with bifurcated drift orbits have, to the best of our knowledge, not been explicitly studied during dropout events. The contribution of these particles to the total dropout is therefore unknown.

In this study, we evaluate the effect of the geomagnetic field treatment to quantify the losses of outer radiation belt electron populations to the magnetopause, using simulated PSD in combination with *K*‐specific LCDS. We use the dropout event that occurred during the October 2012 storm as a case study. This study specifically focuses on outward loss across the magnetopause and so does not consider loss via precipitation. We evaluate the role of radial diffusion when modeled over a dipolar and non‐dipolar geomagnetic field to the loss across the magnetopause, and additionally examine the effect of the treatment of magnetic field lines with local maxima on the loss calculation. We evaluate the loss to the magnetopause with no radial diffusion (so only magnetopause shadowing contributes to loss), radial diffusion with the empirical Brautigam and Albert (2000) model that assumes a dipolar geomagnetic field, and radial diffusion with the event‐specific Cunningham (2016) model evaluated over a non‐dipolar geomagnetic field. We additionally evaluate the contribution of Shabansky type 1 particles to loss during this storm, which provides insight into the loss of particles undergoing true drift orbit bifurcation during geomagnetic storms.

## 2 DataSets and Methods

### 2.1 DataSets

The Van Allen Probes (RBSP, Mauk et al. [2012]) are twin probes on highly elliptical orbits with an orbital period of approximately 9 hr. Van Allen Probes A and B were launched on 30 August 2012 and were deactivated on 18 October 2019, and 19 July 2019, respectively. The Van Allen Probes were equipped with the Magnetic Electron Ion Spectrometer (MagEIS, Blake et al. [2013]) and the Relativistic Electron‐Proton Telescope (REPT, Baker et al. [2013]), which measured the electron fluxes over a wide energy range. Electron fluxes can also be obtained from the Global Positioning System (GPS, Morley et al., 2017). This is a constellation of satellites in inclined circular orbits at a radial distance of 4.2 *R*_*E*_ and orbital periods of 12 hr that measure electron counts with either the Combined X‐ray and Dosimeter (CXD, Tuszewski et al. [2004]) or the Burst Detector Dosimeter for Block II‐R (BDD‐IIR; Cayton et al. [1998]) instruments. GPS data is publicly available from December 2000 to November 2020, and this data product includes electron fluxes evaluated at selected energies that were calculated from the electron counts. These fluxes were calculated using a flexible forward model and then cross‐calibrated against Van Allen Probes measurements (Morley et al., 2016). Solar wind and IMF data were taken from the OMNI database in 1 hr time resolution. The Disturbance Storm Time (Dst) data and the planetary *K*_*p*_‐index data are retrieved from WDC Kyoto in one and 3 hr time resolutions, respectively.

The three‐dimensional Dynamic Radiation Environment Assimilation Model (DREAM3D) Fokker‐Planck code was used to simulate the electron PSD during the October 2012 dropout event. The one‐dimensional DREAM simulation evolved out of a desire to nowcast the energetic electron environment by coupling a 1D radial diffusion physics model to spacecraft observations using data assimilation (Reeves et al., 2012). DREAM3D uses the same approach to convert spacecraft observations to PSD for use as initial and boundary conditions in DREAM, but DREAM3D simulation output is not merged with the spacecraft data in a data assimilative framework. Other examples of Fokker‐Planck codes include those described by Glauert et al. (2014); Ma et al. (2015); Subbotin and Shprits (2009); Varotsou et al. (2005). Multiple transport and loss processes can be modeled with DREAM3D (Tu et al., 2013, 2014), although we removed pitch‐angle and momentum diffusion due to interactions with very‐low frequency waves from all simulations. This ensured that radial diffusion was the only non‐adiabatic transport process acting in the simulation and additionally ensured that loss to the magnetopause was the only permanent loss process. An open outer boundary in L* was specified at L* = 11 with *δf*/δL* = 0, in the same way as in Tu et al. (2014); Tu et al. (2019). Data from the Van Allen Probes were used to initialize the electron PSD profiles.

We used the DREAM3D simulated PSD in combination with the LANLGeoMag LCDS code (Albert et al., 2018; Henderson et al., 2018) in order to evaluate loss to magnetopause. For a given pitch angle (determined from input *K*) and spatial location, the LCDS model determines the magnetic mirror point (*B*_*M*_) on a single field line. The second adiabatic invariant and *B*_*M*_ values are then used as targets to identify magnetic field lines on the same drift shell at each magnetic local time (MLT). If a closed drift shell is identified, the corresponding L* value is calculated; this closely follows the prescription outlined in Roederer and Lejosne (2018) and is described by Albert et al. (2018) (Section 2.2). This process is repeated at increasing radial distances until L* is no longer defined, at which point the last closed drift shell can be identified. Drift shells (and hence L*) describe the trajectory of particles at constant *K*. Therefore, the LCDS is also dependent on *K* and can vary significantly for different values of *K* (e.g., Tu et al., 2019). The LANLGeoMag LCDS can be evaluated with different treatments of field lines with local maxima. We evaluate two settings here: first rejecting all field lines with local maxima when constructing drift shells (i.e., only including drift shells corresponding to non‐Shabansky particles) and then allowing field lines with a local maxima less than *B*_*M*_ in drift shell construction (including drift shells that Shabansky type 1 particles would travel along). Drift shells corresponding to Shabansky type 2 and 3 orbits are rejected when evaluating the LCDS with both of these settings.

### 2.2 Method

We simulated the October 2012 dropout event with three radial diffusion models. The evaluated radial diffusion settings were (a) no radial diffusion, (b) radial diffusion with the empirical Brautigam and Albert (2000) model, and (c) radial diffusion with the event‐specific, non‐dipolar Cunningham (2016) model. The Brautigam and Albert (2000) model is an empirical model of radial diffusion determined from stormtime Combined Release and Radiation Effects Satellite (CRRES) measurements that were fit to the theoretical L‐shell dependency provided by Fälthammar (1965) and Schulz and Lanzerotti (1974). This model evaluates transport over L‐shell, so implicitly assumes that radial diffusion occurs over a dipolar background field (which is also assumed in Fälthammar [1965]; Schulz & Lanzerotti [1974]), and has the 3‐hr *K*_*P*_ index as the sole time‐varying input. The Brautigam and Albert (2000) model were generalized to off‐equatorial populations following the theory outlined in Sections III.2 and III.3 of Schulz and Lanzerotti (1974). The Cunningham (2016) model evaluates radial diffusion in adiabatic space with an arbitrary geomagnetic field by determining the changes in *K* and L* in response to global electromagnetic fluctuations. Here we use the TS04 geomagnetic field model, which includes the asymmetry between the dayside and nightside of the magnetosphere due to solar wind compression, in addition to other key factors that distort the Earth's magnetic field from the dipole assumption (Tsyganenko & Sitnov, 2005). Cunningham (2016) uses the *K*_*p*_‐dependence of the total ULF wave power from Brautigam and Albert (2000) and the frequency‐dependence of the ULF wave power spectral density assumed by Fälthammar (1965) but applies the quasilinear theory to an arbitrary background magnetic field model following the approach of Schulz and Lanzerotti (1974). Radial diffusion coefficients obtained from the Brautigam & Albert and Cunningham models are referred to as *D*_*LL*_(*B&A*) and *D*_L*L*_(*C*) respectively. The *D*_L*L*_(*C*) were output from the DREAM3D simulation at 30 min time intervals, while the *D*_*LL*_(*B&A*) were calculated from Equations 4 and 6 in Brautigam and Albert (2000) and has 3 hr time resolution due to the *K*_*p*_ parametrization. We selected these two radial diffusion models in order to compare the treatment of the geomagnetic field as either dipolar or non‐dipolar to transport via radial diffusion across the magnetopause. The simulation with no radial diffusion additionally allows evaluation of the loss to the magnetopause solely due to compression of the magnetopause.

Each radial diffusion setting was evaluated for two drift orbit construction criteria in DREAM3D, referred to here as non‐Shabansky and Shabansky 1 treatment, for a total of six simulations. With the non‐Shabansky treatment, drift orbits were solely constructed from magnetic field lines with a single minima. In other words, Shabansky particles of types 1, 2, and 3 were not included in the PSD distribution. The simulations with Shabansky 1 treatment allowed magnetic field lines with multiple magnetic field minima in the drift shell construction if the local maxima were less than the magnetic mirror value. In this case, Shabansky type 1 orbits had a defined L* and thus were included in the PSD profile while Shabansky type 2 and 3 particles were excluded from the PSD profile. We do not attempt to compute L* for particles undergoing drift orbit bifurcation, so Shabansky type 2 and 3 particles are not included in any simulations. The drift shells of Shabansky type 1 electrons in these simulations are always at greater L* than the drift shells of non‐Shabansky electrons with given *μ* and *K* values due to the strong Chapman‐Ferraro current at the magnetopause that drives the equatorial maxima in the magnetic field. The evaluation of Shabansky type 1 particles provides insight into the contribution of particles experiencing a magnetic field with local maxima at the equator to the total loss across the magnetopause during dropout events, which provides an indication of the contribution of Shabansky type 2 and 3 particles to these events.

The loss calculation was performed using PSD taken from each of the DREAM3D simulations in combination with the LANLGeoMag LCDS. The PSD was taken at a pre‐storm time, denoted as *t*_*i*_. We then determined the minimum value of LCDS during the dropout event for each evaluated *K* value, denoted as minLCDS, and defined the time that minLCDS occurred as *t*_*f*_. The initial PSD in a given initial L* bin $\left(L_{i}^{*}\right)$ is diffused to final L* bin at time *t*_*f*_ $\left(L_{f}^{*}\right)$ by

$$L_{i}^{*}+\sum_{t_{i}}^{t_{f}}\langle \Delta L^{*}\rangle =L_{f}^{*},$$

where 〈ΔL*〉 is the amount of net transport in adiabatic space that the population underwent due to radial diffusion in time Δ*t*. This was calculated based on the radial diffusion coefficients of each model, according to

$$D_{L*L*}=\frac{1}{2}\frac{{\langle \Delta L^{*}\rangle }^{2}}{\Delta t}.$$

The total change in L* between time *t*_*i*_ and *t*_*f*_ was found iteratively under the assumption that radial diffusion always acted to diffuse particles radially outward. This assumption of purely outward net diffusion was made in order to determine the maximum possible loss that occurs during dropout events; we note that the direction of net transport during radial diffusion is dependent on the gradient of the PSD, but inward diffusion is not evaluated here as it does not contribute to losses across the magnetopause. In this approach, the radial diffusion coefficient at initial location $L_{i}^{*}$ was used to calculate $\langle \Delta L_{\Delta t_{1}}^{*}\rangle$, which is the net amount of diffusion in adiabatic space that occurred during the first timestep (Δ*t*_1_). At the next timestep (Δ*t*_2_), the radial diffusion coefficient was calculated at $L^{*}=L_{i}^{*}+\langle \Delta L_{\Delta t_{1}}^{*}\rangle$ to compute the net change in L* during the second timestep $\left(\langle \Delta L_{\Delta t_{2}}^{*}\rangle \right)$, and so on until time *t*_*f*_ was reached. This was done to account for the L* dependence in the radial diffusion coefficients, which results in different amounts of diffusion occurring for different initial L*. We set Δ*t* = 0.5 hr for the Cunningham (2016) model and Δ*t* = 3 hr for the Brautigam and Albert (2000) model, corresponding to the time resolution provided by the DREAM3D outputs and the *K*_*p*_ parametrization respectively.

We define loss to the magnetopause for the purposes of this study as the amount of the initial PSD distribution that is transported beyond minLCDS between the *t*_*i*_ and *t*_*f*_. There is some discrepancy between the outer boundary of DREAM3D and the LANLGeoMag LCDS value (see Figure A2), which arises due the different methods for drift shell construction in the two models. We have set the *D*_L*L*_(*C*) value as 0 days^−1^ at L* beyond the DREAM3D outer boundary, as the calculation of *D*_L*L*_(*C*) requires a closed drift shell. The LANLGeoMag LCDS however closely follows the DREAM3D outer boundary until time *t*_*f*_ for each evaluated *K* so, while this treatment of *D*_L*L*_(*C*) going to 0 days^−1^ immediately below the magnetopause is unphysical, we expect the introduced error to be minor during the storm phase evaluated in this study.

In order to determine the $L_{i}^{*}$ values corresponding to loss across the magnetopause, we define parameter $L_{\mathrm{loss}}^{*}$ as

$$L_{\mathrm{loss}}^{*}+\sum_{t_{i}}^{t_{f}}\langle \Delta L^{*}\rangle =\text{minLCDS},$$

which depends on *K* due to the *K*‐dependency of the LCDS, in addition to depending on the choice of the radial diffusion model. This means that electrons initially located at $L_{i}^{*}$ = $L_{\mathrm{loss}}^{*}$ are transported to final L* equal to the minLCDS, so are the most distant electrons in the prestorm PSD distribution that remain trapped within the magnetopause. For the scenario with no radial diffusion, no transport across L* takes place so $L_{\mathrm{loss}}^{*}$ = minLCDS. Therefore, any PSD at initial L* bin greater than $L_{\mathrm{loss}}^{*}$ are lost to the magnetopause, while PSD initially located at $L_{i}^{*}\leq L_{\mathrm{loss}}^{*}$ remain trapped within the magnetopause after undergoing radial diffusion. We therefore define the lost PSD as

$$\text{Lost}\;\text{PSD}=\sum_{L^{*}}\text{PSD}\left(t_{i};\;L^{*}>L_{\mathrm{loss}}^{*}\right).$$

We evaluated the loss for three *K* values, 0.075, 0.1, and 0.125 *R*_*E*_ *G* ^1/2^, which correspond approximately to 40–50° equatorial pitch angle at RBSP apogee immediately prior to the October 2012 storm. For each *K* value considered in this study, we calculate the loss experienced by electron populations with a range of *μ* values corresponding to relativistic energies. This allows us to evaluate the contribution of various factors to the loss across the magnetopause experienced by a wide range of populations during this dropout event. In reality, a smaller percentage of electrons at a given initial L* will reach the magnetopause (and hence be lost) when they need to diffuse across a larger distance due to various other processes taking place in the radiation belt that may interfere with the particles' outward radial transport. We additionally do not include loss after the time of minLCDS in our calculations, although the loss to the magnetopause would likely continue past this time due to continuing outward radial diffusion. This method of calculating loss to the magnetopause, therefore, provides an upper limit to the loss experienced by a given population in a given time and does not attempt to reproduce the losses measured by satellite observations. Instead, the objective of this study is to make idealized assumptions about the Earth's geomagnetic field in order to evaluate the effect that these assumptions have on the electron losses during dropout events.

## 3 Results

### 3.1 Overview of October 2012 Geomagnetic Storm

We selected the dropout event that occurred during 8–9 October 2012, storm to evaluate the role of the treatment of the Earth's geomagnetic field to loss across the magnetopause. This storm was one of the first of the Van Allen Probes era and has been extensively studied, for example, by Kress et al. (2014); Kurita et al. (2016); Pokhotelov et al. (2016); Reeves et al. (2013); Thorne et al. (2013); Tu et al. (2014). A summary of this storm and its impact on radiation belt electron fluxes is presented below.

**Figure 1:**
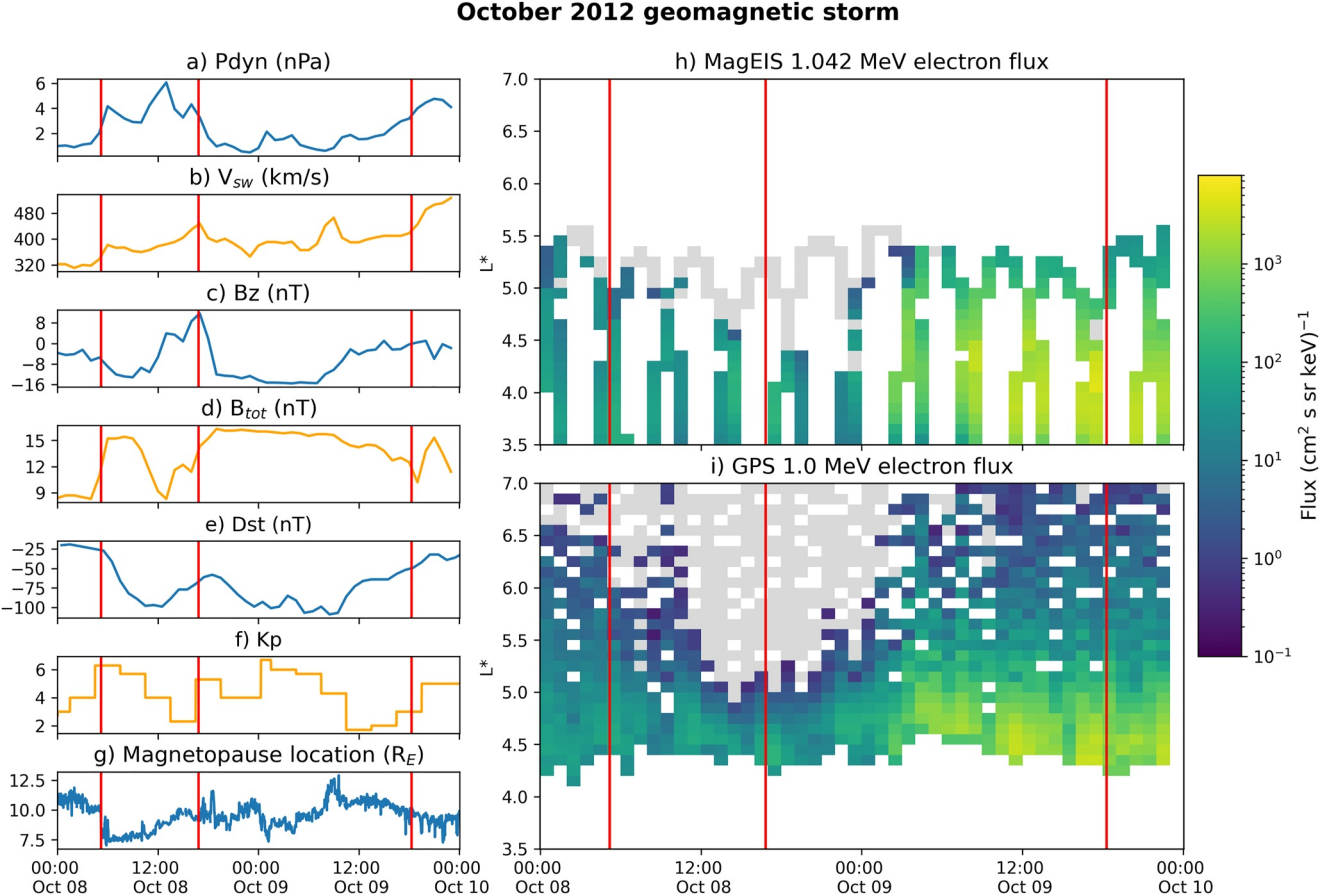
Summary of the solar wind and interplanetary magnetic field (IMF), geomagnetic indices, magnetopause location, and electron flux response for October 8–9, 2012. The sheath and ejecta timings are represented by vertical red lines in each subplot. Subplots a – d show the dynamic pressure, solar wind velocity, z component, and total IMF respectively. Subplots e and f show the Dst and *K*_*p*_ geomagnetic indices, and subplot g shows the magnetopause location. Subplots h and i show the flux response of the 90°, 1 MeV electron population, with subplot h showing combined MagEIS measurements from RBSP‐A and RBSP‐B and subplot i showing combined data from 11 GPS satellites. Gray boxes in subplots h and i show bins where at least one satellite was present but zero flux was recorded, while bins with no satellites present are white. See Figure A1 for further details of the orbital coverage by the Van Allen Probes and GPS during this event.

The October 2012 geomagnetic storm was driven by an interplanetary coronal mass ejection (ICME) with distinct sheath and ejecta phases (type 3 according to the classification system outlined in Kilpua et al., 2015). The ICME sheath impacted the magnetosphere at ∼05:12 UT, 8 October, and the leading edge of the ejecta was detected at ∼16:50 UT, 8 October. The trailing edge of the ejecta reached the magnetopause at ∼18:17 UT, 9 October (key times taken from Nieves‐Chinchilla et al. [2018] and timeshifted to the Earth's bow shock nose). Figure 1 gives an overview of this event, with subplots 1a–d showing key solar wind parameters, subplot e showing the 1‐hr Dst index and subplot f showing the 3‐hr *K*_*p*_ index. Figure 1g shows the subsolar magnetopause location during this storm calculated from the Shue et al. (1998) magnetopause model. The response of relativistic electron fluxes is shown in the right panels of Figure 1. Figure 1h shows the 1 MeV equatorial electron fluxes measured by MagEIS during the storm. The pre‐calculated flux data product from the 11 GPS satellites with active CXD instruments during this event is shown in Figure 1i. Electron fluxes measured by both MagEIS and GPS are binned over 1 hr and 0.1 L*, with L* calculated using the TS04 model.

There is a clear “double dip” in Dst, with the minima occurring at ∼12 UT on October 8 during the sheath and at ∼9 UT on October 9 during the ejecta phase. The *K*_*p*_ index also has two distinct peaks, becoming elevated at approximately the same times as Dst reaches its minima. The low (minimum of −102 nT) Dst values and high (6+) *K*_*p*_ values are signatures that this was a moderate geomagnetic storm. The *B*_*z*_ component is strongly southward during the leading portion of the sheath phase and during the first half of the ejecta. Additionally, the dynamic pressure is elevated throughout the sheath, so the solar wind conditions are favorable during the sheath to cause significant magnetopause compression. This is reflected in the magnetopause location, which is compressed to ∼7.5*R*_*E*_ during the first half of the sheath and then recovers throughout the latter half of the sheath to approximately 10 *R*_*E*_ by the time of ejecta impact.

**Figure A1:**
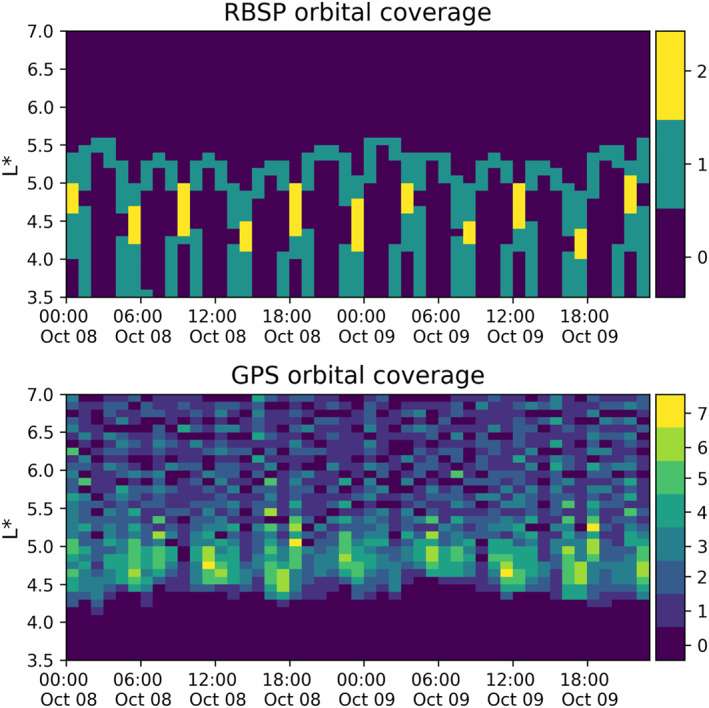
Orbital coverage of the Van Allen Probes (top) and global positioning system (bottom) satellites during the October 2012 dropout event. The color scale shows the number of satellites in each time/L* bin, for bins of 0.1 L* and 1 hr.

The Van Allen Probes measurements show a flux depletion that begins during the sheath and moves to lower L* during the early stages of the ejecta. The GPS data in turn shows that the fluxes disappear completely at L* > ∼5 and decrease at lower L* between 8 October ∼11 UT and 9 October ∼5 UT. There is consistent coverage by the RBSP and GPS satellites during this time (as shown by the gray boxes in subplots 1h‐i and Figure A1 that indicate when zero fluxes were recorded), revealing that the dropout was so intense that the outer radiation belt electrons were depleted below the instrument levels of both MagEIS and CXD. We, therefore, conclude that there is a total depletion of relativistic electron fluxes at L* > ∼5 and a partial depletion at lower L* that started around the mid‐sheath and persisted for over 12 hr.

Sheath regions are associated with enhanced losses across the magnetopause due to the combined effect of enhanced magnetopause compression and increased ULF‐driven radial diffusion (Hietala et al., 2014; Kalliokoski et al., 2020). A simulation performed by Hudson et al. (2014) found that loss to the magnetopause was the dominant contributor to the October 2012 dropout, as evidenced by the energy and pitch‐angle dependence of the electron flux losses, with magnetospheric ULF wave activity being highly elevated as low as L‐shell of 5. Pokhotelov et al. (2016) also found enhanced ULF activity based on satellite observations that resulted in strong radial diffusion during the sheath of this storm, so outward transport of relativistic electrons likely significantly contributed to losses across the magnetopause. The intense dropout of relativistic electrons did not begin at the same time as the strongest magnetopause compression, although, as discussed earlier, the LCDS provides a more useful indicator of the loss of a specific electron population across the magnetopause than the spatial magnetopause location.

### 3.2 Last Closed Drift Shell

**Figure 2:**
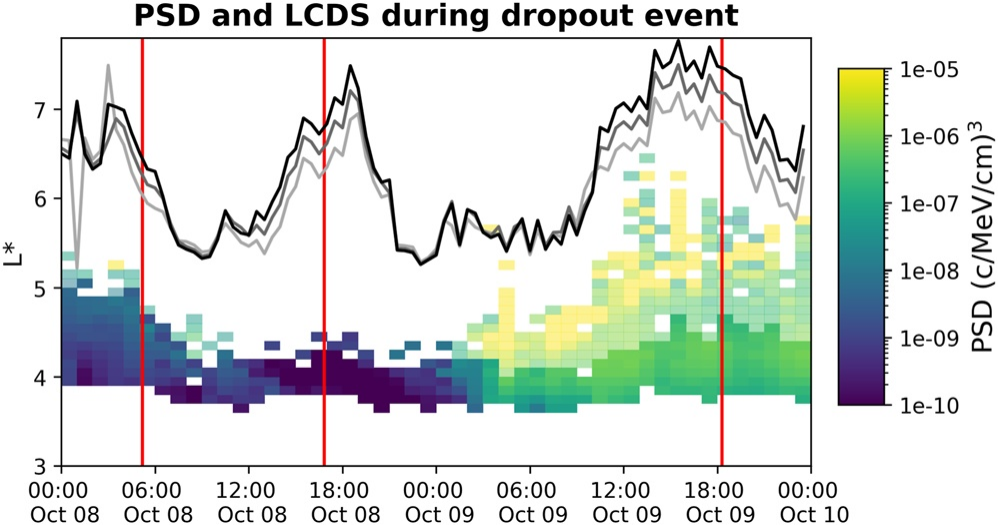
Last closed drift shell (LCDS) during the dropout event in comparison to relativistic electron phase space density (PSD). The LCDS is shown for three *K* values with drift shell construction that rejects all bifurcated magnetic field lines. Light gray corresponds to *K* = 0.075 *R*_*E*_ *G* ^1/2^, dark gray corresponds to *K* = 0.1 *R*_*E*_ *G* ^1/2^ and black corresponds to *K* = 0.125 *R*_*E*_ *G* ^1/2^. For comparison, we show the PSD calculated from the GPS electron counts for the population with *K* = 0.1 *R*_*E*_ *G* ^1/2^ and *μ* = 2300 MeV/G, and this population is typical of the relativistic electron PSD response. PSD is binned over 0.1 L* and 1 hr. The semi‐transparent portion of the GPS PSD colormap indicates bins where the pitch angle distribution ratio was less than 0.1, that is, bins where the data represents less than 10% of the total population. The PSD magnitude in the semi‐transparent bins is not representative of the entire radiation belt population but it is shown here as an indication of the spread of radiation belt electrons over L*. The sheath and ejecta timings are represented by vertical red lines.

The LCDS for electron populations with *K* = 0.075, 0.1, and 0.125 *R*_*E*_ *G* ^1/2^ are shown in Figure 2, as calculated from the LANLGeoMag LCDS code. Figure 2 shows a comparison between the LCDS with the non‐Shabansky treatment to GPS PSD for the *K* = 0.1 *R*_*E*_ *G* ^1/2^, *μ* = 2,300 MeV/G population. The PSD was calculated from the GPS counts by first implementing the flux forward model to obtain the fluxes (Morley et al., 2016), which was then utilized in combination with the empirical relativistic electron pitch angle distribution model (REPAD, Chen et al., 2014) to compute the PSD according to the procedure outlined in Hartley and Denton (2014). The population shown in Figure 2 had a response that was typical for relativistic electron populations during this dropout event. The LCDS values for each *K* value are significantly lower than the spatial location of the magnetopause calculated according to the Shue et al. (1998) model (Figure 1g), consistent with Matsumura et al. (2011).

The LCDS results trend similarly for the three evaluated *K* values and the value of LCDS at a given time generally increases with increasing *K* (i.e., smaller equatorial pitch angles), which occurs due to drift shell splitting. The LCDS is at L* of ∼6.5–7 prior to the ejecta impact, and it drops to approximately L* = 5.5 within a few hours of sheath impact for each *K*. The PSD at L* = 4.5–5.5 is rapidly and totally depleted following this decrease in LCDS, and the PSD at lower L* (∼4) becomes strongly depleted approximately 3 hours after the lowest LCDS values during the sheath phase of the storm. The LCDS is more distant than the greatest L* corresponding to in‐situ observations throughout the decrease in LCDS; this gap corresponds to outward radial transport across the magnetopause, as also observed in Olifer et al. (2018). The LCDS increases significantly in the latter half of the sheath phase, peaking shortly after the time of ejecta impact, with higher *K* values peaking at the greatest LCDS value. This increase in LCDS is concurrent with the recovery of Dst to more moderate values (Figure 1e). The PSD does not recover during this period of high LCDS values and increased Dst, either in magnitude or L* spread, showing that the dropout during the sheath phase was a non‐adiabatic loss that the belts did not yet recover from.

The LCDS decreases again when the ejecta impacts the magnetosphere, remaining at low values until the trailing portion of the ejecta in the early hours of October 9, with minimal *K* dependency during this time. The LCDS and PSD both recover during the latter half of the ejecta, with LCDS increasing for all *K* and PSD spreading to higher L* and intensifying. The concurrent LCDS increase and PSD enhancement indicates that some process (possibly local acceleration, Reeves et al. [2013]; Thorne et al. [2013]; Tu et al. [2014]) acted to enhance the PSD at lower L*, which were then transported outward to refill the previously open drift shells. An alternative explanation for this enhancement is the inward radial diffusion of particles from the outer boundary of the radiation belts, similar to the event studied in Ozeke et al. (2019), which is supported by the peak GPS PSD occurring at high L*. We however note that these peak PSD at high L* are measured when the GPS is at such high latitudes that they sample less than 10% of the total population (indicated by the semi‐transparent bins), making it difficult to conclusively state the dominant mechanism during the storm recovery from these data.

We calculate the loss across the magnetopause using the minimum LCDS value for each *K* value that occurred during the dropout. The LCDS reach their lowest value throughout the entire storm at 23:00 on 8 October, soon after the ejecta impact, but it is clear from the in‐situ measurements of the electron populations that the depletion began during the sheath portion of the storm. The minimum LCDS during the sheath region (hereafter referred to as minLCDS) is ∼0.07–0.09 larger than the LCDS values at 23:00, 8 October, depending on the *K* value. The minLCDS for *K* = 0.075 *R*_*E*_ *G* ^1/2^ is 5.38 and occurs at 13:00, 8 October, while the minLCDS for *K* = 0.1 and 0.125 *R*_*E*_ *G* ^1/2^ both occur at 9:00, 8 October, reaching values of 5.35 and 5.32, respectively. These timings are consistent with the dropout observed in the electron flux/PSD measurements and the timing of the first Dst minima, and demonstrate a trend of decreasing minLCDS with increasing *K* value.

The results of Albert et al. (2018) were used to estimate the error in the minLCDS. Albert et al. (2018) studied the LCDS calculated by different models during four events, and Event 1 of this study was a storm in March 2013 that first caused a depletion of relativistic electrons and then a strong enhancement of electron fluxes (Baker et al., 2014). This is qualitatively similar to the October 2012 event, and the LCDS for *K* = 0.11*R*_*E*_ *G* ^1/2^ (shown in Figure 4 of Albert et al. [2018], with the same geomagnetic field model as in this study) demonstrates a “double dip” that is similar to the results shown in Figure 2 of this study. The minLCDS obtained from these alternate models range from L* of approximately 5–5.5, with the LANLGeoMag model producing the approximate median value during the March 2013 event. We therefore take our uncertainty in the minLCDS obtained from LANLGeoMag as ± 0.25, from which we obtain our uncertainty in $L_{\mathrm{loss}}^{*}$ and the calculated loss. Evaluation of the error in *D*_*LL*_(*B&A*) and *D*_L*L*_(*C*) is beyond the scope of this study, so the contribution of uncertainty in the radial diffusion coefficients is not included in our error analysis.

A comparison between LCDS calculated with non‐Shabansky and Shabansky 1 treatments is shown in Figure S1 of Supporting Information S1; these treatments produce nearly identical LCDS, which is consistent with the LCDS results for four events evaluated in a study by Albert et al. (2018). This occurs because the current LANLGeoMag algorithm often fails to converge to the expected tolerance on Shabansky type 1 orbits, thus marking them as undefined. The LANLGeoMag LCDS results, therefore, do not vary significantly with the treatment of bifurcated drift orbits. The same error of ±0.25 is taken for the minLCDS with the Shabansky 1 treatment as in the non‐Shabansky treatment.

### 3.3 Electron Loss

**Figure 3:**
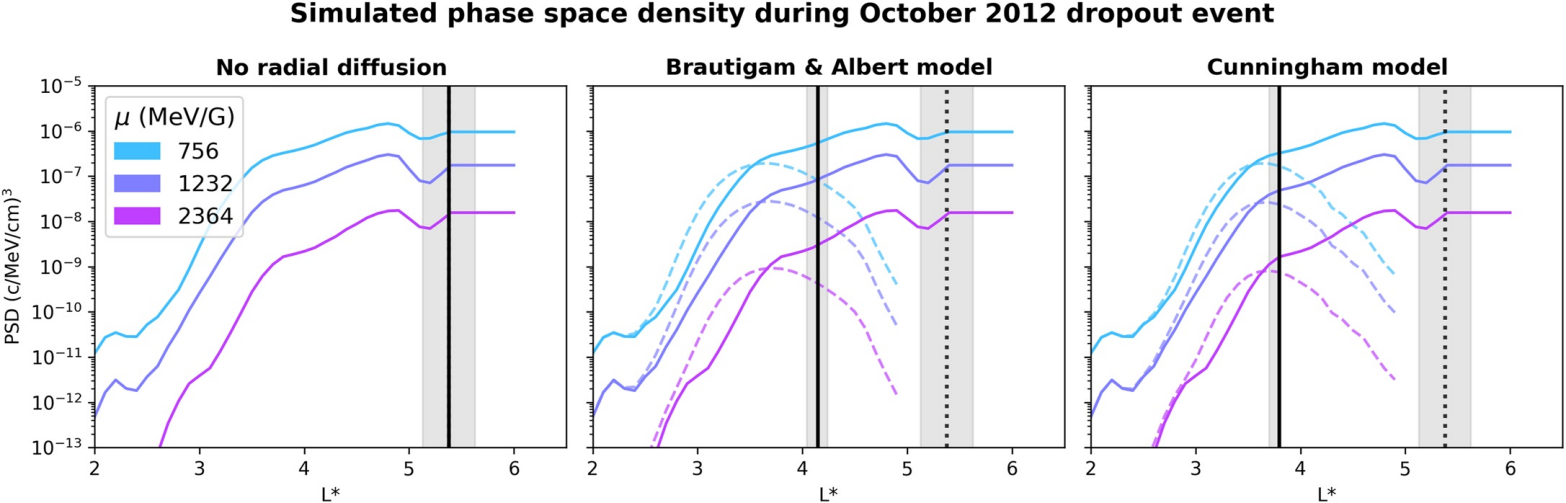
Phase space density (PSD) of electrons with *K* = 0.1 *R*_*E*_ *G* ^1/2^ and selected *μ* corresponding to relativistic energies, with the plot color corresponding to the *μ* value. The PSD is taken from the Dynamic Radiation Environment Assimilation 3D simulation, with solid lines showing the pre‐storm PSD, taken at 00:15 October 8, and dashed lines showing the PSD at 09:15 October 8, which is the time that the minimum last closed drift shell occurred during the dropout for this *K* value. Each subplot corresponds to a different radial diffusion criterion. The dotted vertical lines show the minimum LCDS value (which is the same for each radial diffusion model) and solid vertical lines show $L_{\mathrm{loss}}^{*}$ for a given radial diffusion criterion. The shaded regions around minLCDS and $L_{\mathrm{loss}}^{*}$ indicate the error in these values.

We begin by presenting the results for the electron populations composed solely of non‐Shabansky particles using a pre‐storm PSD profile from the DREAM3D simulation. The pre‐storm PSD profile was taken at 00:15 UT, October 8, approximately 5 hr before the ICME shock arrived at the magnetopause, for three *K* values (0.075, 0.1, and 0.125 *R*_*E*_ *G* ^1/2^) and a wide range of *μ* that correspond to relativistic energies at geostationary orbit. The initial PSD for selected populations is shown in Figure 3. The PSD at *t*_*f*_ was also taken from DREAM3D for each of the radial diffusion criteria and is shown in Figure 3, with each subplot showing the PSD evaluated with a different radial diffusion model. The PSD distribution at *t*_*f*_ is not visible in the subplot showing the simulation results with no radial diffusion because no processes occur that could change the PSD distribution over L*. The minLCDS and $L_{\mathrm{loss}}^{*}$ corresponding to each radial diffusion criterion are shown by vertical lines in Figure 3.

Any of the initial PSD to the right of the line representing $L_{\mathrm{loss}}^{*}$ in a given subplot is lost to the magnetopause and is summed to give the total loss to the magnetopause experienced by a given population. When radial diffusion is enabled in the simulation, we observe that some of the final PSD distribution is located at $L_{f}^{*}$ beyond $L_{\mathrm{loss}}^{*}$. This occurs because $L_{\mathrm{loss}}^{*}$ is the L* beyond which the *initial* PSD is lost to the magnetopause due to outward transport via radial diffusion between the initial time and time of minLCDS. Some of the *final* PSD distribution will then be located at $L_{\mathrm{loss}}^{*}<L^{*}<$minLCDS without being lost to the magnetopause. For example, initial PSD that is located at $L^{*}=L_{\mathrm{loss}}^{*}$ will be transported outward to final L* that is greater than $L_{\mathrm{loss}}^{*}$ but less than minLCDS, so is not lost to the magnetopause in the evaluated timeframe and is not included in the loss calculation.

**Figure A2:**
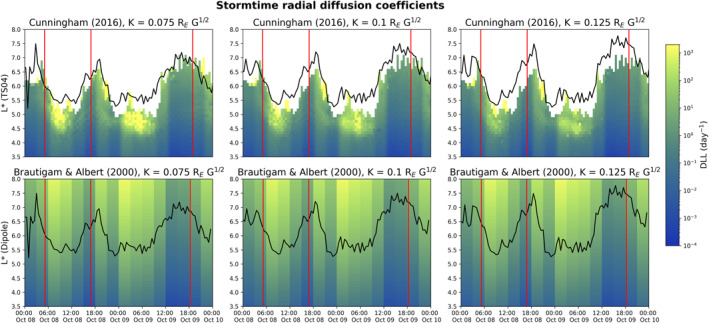
Radial diffusion coefficients obtained with the Cunningham (2016) model (top row) and Brautigam and Albert (2000) model (bottom row) during the October 2012 geomagnetic storm. The vertical red lines show the timings of the arrival of the sheath, ejecta leading edge, and ejecta trailing edge of the interplanetary coronal mass ejection driving this storm. The overlaid black line shows the LANLGeoMag last closed drift shell (LCDS) results for the three *K* values, with drift orbit construction criteria that reject all magnetic field lines with local maxima. The L* bins have width of 0.1 in each subplot and the time bins correspond to the time resolution of each radial diffusion model (0.5 hr for Cunningham [2016] and 3 hr for Brautigam and Albert [2000]). The Cunningham (2016) model provides diffusion coefficients at L* bins within the outer boundary of the DREAM3D simulation, which closely follows the LCDS from LANLGeoMag. Radial diffusion coefficients from the Brautigam and Albert (2000) model can be calculated for any L* value so are shown over the full range of plotted L*. The color scale is the same for each subplot, and high radial diffusion coefficients (yellow) correspond to high levels of radial transport.

Table 1 gives the values of $L_{\mathrm{loss}}^{*}$ for each of the *K* values evaluated in this study, with the uncertainty in $L_{\mathrm{loss}}^{*}$ carried through from the ±0.25 uncertainty in the minLCDS. For each *K*, the $L_{\mathrm{loss}}^{*}$ value obtained with *D*_L*L*_(*C*) is lower than with *D*_*LL*_(*B&A*), and both models significantly lower the $L_{\mathrm{loss}}^{*}$ value from the scenario with no radial diffusion. Transport according to *D*_*LL*_(*B&A*) gives $L_{\mathrm{loss}}^{*}$ values that are essentially constant across *K*; this indicates that the *K*‐dependence of the Brautigam and Albert (2000) radial diffusion model is just enough to offset the *K*‐dependence of the LCDS results. By contrast, *D*_L*L*_(*C*) results in $L_{\mathrm{loss}}^{*}$ that increases with increasing *K*. This occurs because *D*_L*L*_(*C*) values are higher (corresponding to stronger diffusion) at lower *K*, and the *K*‐dependence of this radial diffusion model is so strong that it overwhelms the opposite *K*‐dependence of the LCDS. The radial diffusion coefficients obtained with the two models for each evaluated *K*‐value during the dropout can be seen in Figure A2. The error range in $L_{\mathrm{loss}}^{*}$ generally decreases as the contribution of radial diffusion to loss across the magnetopause increases, as shown in Table 1. This is because both the radial diffusion coefficients and LCDS are used to calculate $L_{\mathrm{loss}}^{*}$, and radial diffusion becomes increasingly more important during this calculation as outward transport becomes stronger. As mentioned earlier, we do not evaluate the error in the radial diffusion coefficients in this study, although quantifying this uncertainty in radial diffusion models is an important avenue for future study. The variation in $L_{\mathrm{loss}}^{*}$ between radial diffusion models shows that evaluating loss to the magnetopause with radial diffusion evaluated over a non‐dipolar field model results in the dropout penetrating deeper into the heart of the radiation belt than when assuming a dipolar background field, particularly for populations with lower *K* values. The depth at which loss occurs is an important factor to consider when evaluating dropout events, so this is a significant difference between the two radial diffusion models.

**Table 1:** $L_{\mathrm{loss}}^{*}$ for Each *K* Value and Radial Diffusion Criterion Evaluated in This Study

*K* (*R*_*E*_ *G* ^1/2^)	No radial diffusion	Brautigam & Albert model	Cunningham model
0.075	5.13 ≤ 5.38 ≤ 5.63	4.04 ≤ 4.15 ≤ 4.24	3.7 ≤ 3.8 ≤ 3.8
0.1	5.10 ≤ 5.35 ≤ 5.60	4.06 ≤ 4.17 ≤ 4.27	3.9 ≤ 4.0 ≤ 4.1
0.125	5.07 ≤ 5.32 ≤ 5.57	4.06 ≤ 4.17 ≤ 4.27	4.0 ≤ 4.1 ≤ 4.1

*Note*. This is the most distant L* of an electron population that remains trapped during the October 2012 dropout event. The Brautigam and Albert (2000) radial diffusion model is *μ* dependent, and here the $L_{\mathrm{loss}}^{*}$ values are shown as the mean value across all *μ* from 700 to 2,500 MeV/G. The results for the Brautigam & Albert model and no radial diffusion show three significant figures indicating the variation between *K*. The Cunningham diffusion coefficients were obtained from the DREAM3D simulation that was evaluated over an L* grid with a resolution of 0.1, so $L_{\mathrm{loss}}^{*}$ calculated with this diffusion model are presented to two significant figures.

**Figure 4:**
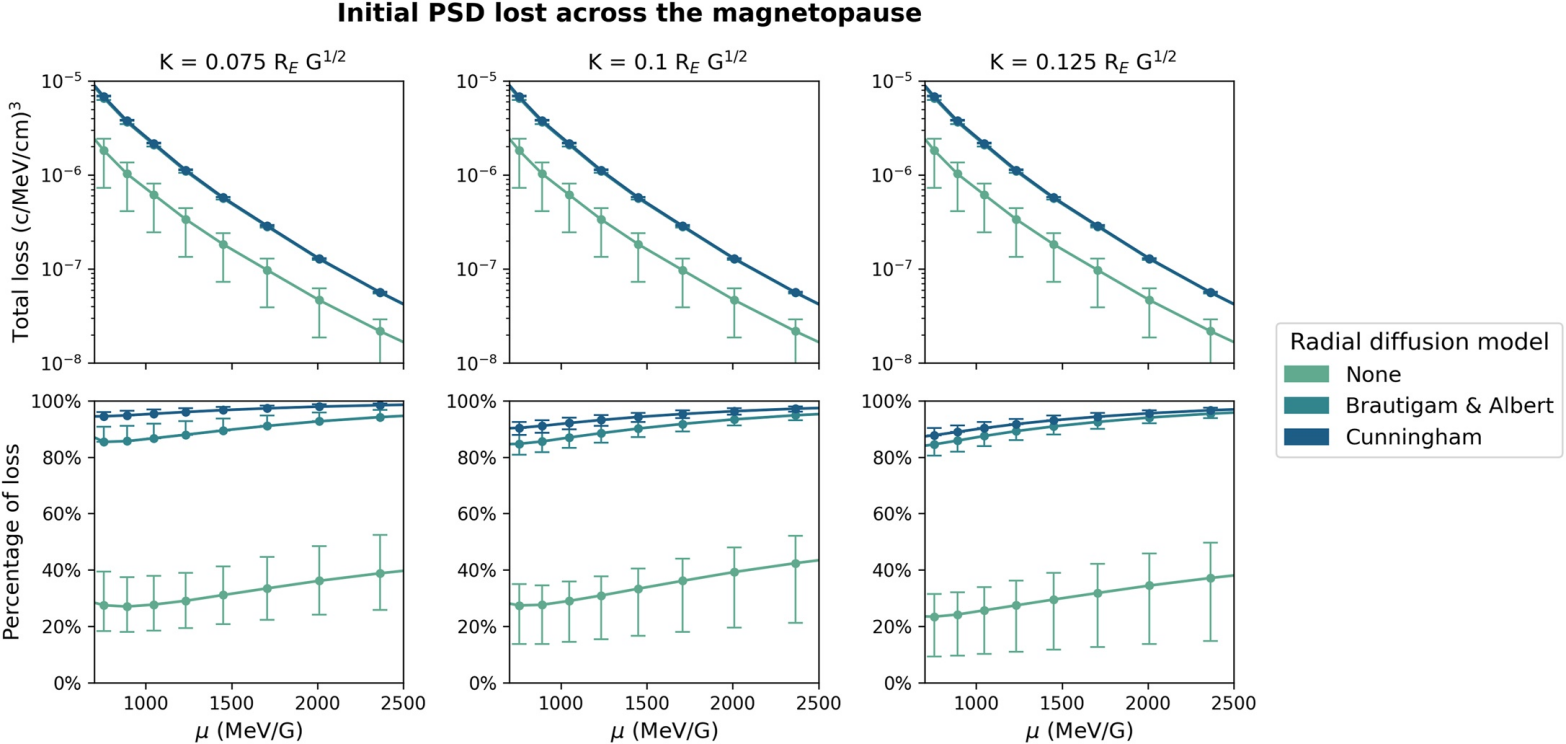
Loss experienced by relativistic electron populations to the magnetopause during the October 2012 geomagnetic storm. The top row shows the total loss calculated by summing the initial phase space density (PSD) at L* beyond $L_{\mathrm{loss}}^{*}$, and the bottom row shows the percentage of the initial population that was lost to the magnetopause. The columns correspond to the three evaluated *K* values. The radial diffusion setting is represented by color, with green showing loss with no radial diffusion (loss purely due to magnetopause compression), while mid and dark blue show loss with the Brautigam and Albert (2000) and Cunningham (2016) radial diffusion models respectively. The error bars are calculated from the uncertainty in the min last closed drift shell of ±0.25, which produces the subsequent uncertainty in both $L_{\mathrm{loss}}^{*}$ and loss in PSD.

The total losses to the magnetopause experienced by relativistic electron populations during this dropout are shown on the top row of Figure 4. The amount of loss experienced by a given population is heavily dependent on *μ* due to differences in the initial size of the given population; the greatest losses occur in the populations with lowest *μ* for a given *K* value simply because there are more electrons available to be lost. We can immediately see that the total loss experienced by a given population is significantly greater when radial diffusion is enabled in the simulation than when no radial diffusion occurs, highlighting the importance of including transport across the magnetopause when evaluating dropout events. In order to better see the impact of the radial diffusion model on the calculated loss, we additionally show the percentage of the initial PSD that was lost on the bottom row of Figure 4. The largest percentage of loss of a given population always occurs with *D*_L*L*_(*C*), with up to 10% more of the initial population being lost with this radial diffusion model than with *D*_*LL*_(*B&A*). This shows that the empirical, dipolar Brautigam and Albert (2000) model underestimates the radial diffusion during this storm and thus underestimates the magnitude of the dropout.

We observe that the percentage of loss increases with increasing *μ* for both models and that the difference between the loss calculated with the Brautigam and Albert (2000) and Cunningham (2016) models decreases with increasing *μ*. This is a result of the initial PSD distribution during this event, not a feature of the geomagnetic field. The peak in PSD occurs at relatively low L* for the lowest evaluated *μ*, and this peak narrows and shifts to higher L* as *μ* increases. The greatest difference between the loss calculated with the two models occurs at lowest *μ* because a significant amount of PSD is located at L* greater than $L_{\mathrm{loss}}^{*}$ calculated with *D*_L*L*_(*C*) but less than $L_{\mathrm{loss}}^{*}$ calculated with *D*_*LL*_(*B&A*), resulting in more loss with the Cunningham (2016) model than with Brautigam and Albert (2000) model. The shift in PSD peak to higher L* with increasing *μ* then means that these peak PSD values are located beyond $L_{\mathrm{loss}}^{*}$ calculated from both models, so the percentage of loss is more similar at high *μ* values. Physically, this simply means that when the peak of the PSD population is located near the magnetopause, the majority of the population will be lost across the magnetopause, even when the outward transport is underestimated. Figure 4 also shows that the uncertainty in the lost PSD produced from uncertainty in the minLCDS is less important than the variation between radial diffusion models. We also observe that the difference in loss between the two radial diffusion models decreases with increasing *K* for a given *μ* value. This occurs due to the stronger *K*‐dependency in *D*_L*L*_(*C*) than in *D*_*LL*_(*B&A*), which can be seen in Figure A2. The Cunningham (2016) diffusion coefficients decrease strongly with increasing *K*, resulting in less loss at higher *K*, while similar amounts of loss are calculated across *K* with the Brautigam and Albert (2000) model. Therefore, the trends in loss across *μ* for constant *K* are not a result of the radial diffusion model, and thus can not be attributed to the treatment of the geomagnetic field as dipolar or non‐dipolar, while the *K*‐dependence of the loss can be directly attributed to differences in the two radial diffusion models.

#### 3.3.1 Shabansky Type 1 Comparison

Next, we evaluate the role of Shabansky type 1 electrons when evaluating loss to the magnetopause during the dropout event. Shabansky type 1 particles are located at higher L* than non‐Shabansky particles for given *μ* and *K* in these simulations, so their inclusion means that the initial PSD now extends to slightly higher L*. The Shabansky type 1 particles in the initial PSD are located at $L_{i}^{*}$ beyond minLCDS for each population evaluated in this study, so they are always included in the loss calculation. Therefore, the loss calculated from initial PSD composed of non‐Shabansky and Shabansky type 1 particles is greater than the loss of solely non‐Shabansky initial PSD for each population and radial diffusion model evaluated in this study. The LCDS is only slightly affected by the treatment of magnetic field lines with local maxima (see Figure S1 of Supporting Information S1) and the value of minLCDS or $L_{\mathrm{loss}}^{*}$ is not affected by the drift shell construction criteria for any of the evaluated *K* values to the resolution of L* = 0.1. Therefore, the sole impact on loss calculated using this approach is due to the initial PSD extending to higher L*, as any variation in $L_{\mathrm{loss}}^{*}$ occurs at smaller L* resolution than that of the initial PSD provided by DREAM3D.

We compute the loss for the same three *K* values as in the previous section, again by summing the PSD located at $L_{i}^{*}$ beyond $L_{\mathrm{loss}}^{*}$. Figure S2 of Supporting Information S1 shows the initial PSD distribution including Shabansky type 1 particles in relation to the $L_{\mathrm{loss}}^{*}$ values corresponding to the three radial diffusion criteria, in the same way as in Figure 3. The total and percentage losses calculated from initial PSD including Shabansky type 1 particles are shown in Figure S3 of Supporting Information S1, analogous to Figure 4. A greater percentage of loss occurs with *D*_L*L*_(*C*) than with *D*_*LL*_(*B&A*), as was also the case for the purely non‐Shabansky populations, and the same trends across *μ* and *K* are observed. Both radial diffusion models significantly increase the total and percentage of loss to the magnetopause during this event compared to the scenario with no radial diffusion.

We observe an increase in both total and percentage loss in all evaluated populations when comparing populations composed of solely non‐Shabansky particles and populations composed of both non‐Shabansky and Shabansky type 1 particles. For example, in the *K* = 0.075 *R*_*E*_ *G* ^1/2^, *μ* = 1024 MeV/G population composed solely of non‐Shabansky particles, 29.1% of the initial population was lost with no radial diffusion, 88.0% was lost with the Brautigam and Albert (2000) model and 96.0% were lost with the Cunningham (2016) model. The same population (*K* = 0.075 *R*_*E*_ *G* ^1/2^, *μ* = 1024 MeV/G) lost 32.4%, 88.5%, and 96.2% of the initial PSD with no radial diffusion, *D*_*LL*_(*B&A*) and *D*_L*L*_(*C*) respectively when including Shabansky type 1 electrons in the initial PSD. Tables S1 and S2 of Supporting Information S1 give further examples of the losses computed for selected populations corresponding to relativistic energies. The trends in loss between purely non‐Shabansky initial PSD and initial PSD composed of both non‐Shabansky and Shabansky type 1 electrons presented in those tables are representative of the impact of Shabansky type 1 electrons on calculated loss to the magnetopause for a broad range of populations with *μ* corresponding to relativistic energies. On average, the loss of relativistic populations across the magnetopause increases by 0.75% when Shabansky type 1 particles are included in the initial PSD distribution, for simulations that include a radial diffusion model. This is minor compared to the effect of the radial diffusion model on the loss (up to 10% difference between models), although we note that the 0.75% value does not include the contribution of all particles that travel along magnetic field lines with local maxima, that is, Shabansky type 2 and 3 particles.

## 4 Discussion

The October 2012 dropout event has previously been studied by Tu et al. (2014), who found that evaluating the loss to the magnetopause with a dipolar radial diffusion model underestimated both the magnitude of loss and its depth in L* when compared to Van Allen Probes data. At high L*, the PSD simulated with a dipolar radial diffusion model underestimated the observed loss by approximately half an order of magnitude for a relativistic electron population, and the simulated dropout extended only as low as L* ∼ 4.5 while the observed dropout penetrated to L* ∼ 3.5 (Tu et al., 2014). The authors proposed that this may be due to underestimated radial diffusion coefficients that were obtained from a combination of the Brautigam and Albert (2000); Brautigam et al. (2005) models, which both assume a dipolar field. Another dropout event took place in June 2015, which was dominated by magnetopause shadowing (Xiang et al., 2017), and was further studied by Tu et al. (2019). Radial diffusion coefficients obtained from a combination of the statistical Ali et al. (2016) and Liu et al. (2016) models improved the accuracy of the simulated PSD from the Brautigam and Albert (2000) and Brautigam et al. (2005) models, with the combined Ali et al. (2016) and Liu et al. (2016) models closely matching in‐situ observations during the dropout while the Brautigam and Albert (2000) and Brautigam et al. (2005) models overestimated the observed PSD by approximately an order of magnitude at L* = 3 (Tu et al., 2019). This discrepancy between radial diffusion models highlights the importance of accurately evaluating radial diffusion during dropout events, both to determine the magnitude of the dropout and the depth at which the loss occurs.

First, our results show that excluding radial diffusion significantly underestimated the losses during this dropout event. This emphasizes the role that outward transport via radial diffusion has on losses across the magnetopause during dropout events and the necessity of including radial diffusion in simulations of geomagnetic storms. Second, we have demonstrated here that the choice of radial diffusion model has a significant impact on the calculated loss to the magnetopause, with up to 10% more loss occurring with the Cunningham (2016) radial diffusion model than the Brautigam and Albert (2000) model for relativistic populations during this event. This trend was found to be true for every electron population evaluated in this study, which encompasses multiple pitch angles and a wide range of energies corresponding to relativistic electron populations. We note that the loss to the magnetopause is also dependent on the initial PSD distribution; populations that peak lower in L* have more significant differences in loss computed with the two models than populations that peak nearer to the magnetopause. The magnetic field perturbations driving radial diffusion were the same in each model, so we can attribute the differences in calculated loss of a given population primarily to the treatment of the geomagnetic field as dipolar or non‐dipolar. The more realistic treatment of the geomagnetic field with the Cunningham (2016) radial diffusion model means that the losses calculated with this model are more representative of the true losses than the Brautigam and Albert (2000) model. These results demonstrate that the use of a dipolar background field model underestimates the loss to the magnetopause due to the underestimation of the radial diffusion that transports particles across the magnetopause. These results emphasize the importance of evaluating radial diffusion in non‐dipolar geomagnetic fields, especially during dropout events.

The impact of the geomagnetic field model on loss across the magnetopause likely occurs due to the interaction between the Dst effect and radial diffusion. The adiabatic Dst effect transports electrons to greater radial distances while L* remains constant due to the relatively slow enhancement of the ring current. It is commonly assumed in radial diffusion studies (including in both Brautigam & Albert [2000]; Cunningham [2016]) that the driving wave activity increases with increasing radial distance, which follows from the assumptions made in Fälthammar (1965) and Schulz and Lanzerotti (1974). While it is difficult to map ULF Pc5 wave activity due to both the multiple wave sources and the large spatial scale of these waves, the assumption of increasing wave activity with increasing radial distance is supported by statistical studies (e.g., Agapitov & Cheremnykh, 2013; Liu et al., 2016; Sandhu et al., 2021), and in‐situ observations during the October 2012 dropout event also generally show greater power spectral density in the ULF Pc4 and Pc5 ranges when the satellites are nearer to their apogee than at lower radial distances (Pokhotelov et al., 2016). The increasing wave activity with radial distance means that greater wave activity would act on a population at constant L* after the Dst effect has occurred, increasing the amount of radial diffusion that this population would undergo at this L*. Therefore, the radial diffusion coefficients at a given L* would increase during the time period that the Dst effect acts to adiabatically move the population radially outward. However, assuming that the driving activity remains constant at a fixed radial distance, *D*_*LL*_ calculated from L‐shell would remain the same at a given L‐shell while the Dst effect takes place. Although we do not evaluate the Cunningham (2016) model against a dipolar field model here, we note that it produces significantly lower radial diffusion coefficients with a dipolar geomagnetic field than with the T89 model for the same input driving wave activity (Cunningham, 2016), with the difference in radial diffusion coefficients occurring as a direct result of the geomagnetic field treatment. In this study, the Dst effect meant that greater radial diffusion coefficients were obtained at a given L* with the Cunningham (2016) model than in the Brautigam and Albert (2000) model (for L* calculated with the TS04 and dipole models respectively), which caused the loss to penetrate to lower L* with radial diffusion modeled against the non‐dipolar geomagnetic field.

Another key concern when evaluating radial diffusion is the treatment of the driving ULF wave activity. The Brautigam and Albert (2000) and Cunningham (2016) radial diffusion models evaluated in this study both assume the frequency dependence of the ULF wave power spectrum that was first presented in Fälthammar (1965). These works assume that the ULF power density spectrum of the time‐varying perturbation is inversely proportional to the squared drift frequency, which results in radial diffusion coefficients that are independent of energy. This assumption however requires verification from observational data, as was emphasized already in Fälthammar's seminal work. Although a number of statistical ULF field models have been developed from satellite observations (e.g., Ali et al., 2016; Liu et al., 2016; Sandhu et al., 2021) these typically examine a single frequency range (e.g., solely ULF Pc5 waves) so do not enable verification or refutation of the assumed frequency dependence. Ozeke et al. (2014) show that the power spectral density of the magnetic field fluctuations is reasonably well described with an empirical fit to the inverse of the squared frequency, up to frequencies of approximately 8 mHz for a range of geomagnetic activity levels, providing some validation for the Fälthammar (1965) assumption. The power spectral density of the electric field is assumed to have no frequency dependence in Ozeke et al. (2014), although the results of Sandhu et al. (2021); Olifer et al. (2019) suggest that the electric field parametrization of Ozeke et al. (2014) (including the frequency dependence) may need further consideration. A thorough evaluation of the ULF power, spectral density frequency dependence is a major concern that should be addressed in future studies, particularly for radial diffusion driven by electric perturbations, as it is central to determining whether or not radial diffusion can be modeled as an energy‐independent process. Determining the frequency dependence of ULF wave power is additionally necessary for the evaluation of the uncertainty in the radial diffusion coefficients that utilize the Fälthammar (1965) assumption.

Additionally, the modeling of ULF wave activity with the *K*_*P*_ index as the sole time‐varying parameter likely also presents limitations for the evaluation of radial diffusion. This is a rather common parametrization for radial diffusion models (e.g., Brautigam & Albert, 2000; Ozeke et al., 2014), although the solar wind velocity and *B*_*Z*_ orientation are other key parameters that control the ULF wave activity (Dimitrakoudis et al., 2022). The *K*_*P*_ parametrization results in 3‐hr time resolution of the radial diffusion coefficients and this time resolution may result in inaccuracies, particularly during geomagnetic storms. The radiation belt response can be significantly affected by the internal structures of ICMEs driving the storm, such as sheath regions (Kalliokoski et al., 2020) and the magnetic polarity of the ejecta (George et al., 2020), that occur on relatively short timescales. Magnetospheric wave activity is no exception: the ULF wave power can vary by orders of magnitude during different phases of geomagnetic storms (Sandhu et al., 2021). It is therefore possible that significant variation in ULF wave activity occurs in less than 3 hr during dropout events, and changes on these timescales can not be captured by *K*_*p*_ parameterized field models. Further study is needed to evaluate radial diffusion with both a non‐dipolar field model and a more complex treatment of the driving wave activity. This could be done by, for example, combining the geomagnetic field treatment of the Cunningham (2016) model with a statistical ULF field model that goes beyond *K*_*p*_ parametrization, or by implementing the methodology presented by George et al. (2022) that extends on the theory outlined in Lejosne (2019).

The results presented here also show that the treatment of magnetic field lines with local maxima at the equator affects the calculated loss to the magnetopause. The inclusion of Shabansky type 1 particles in the initial PSD profile of relativistic populations increased the calculated loss to the magnetopause by an average of 0.75% for a given radial diffusion model, as compared to when only non‐Shabansky electrons are evaluated. This increased loss occurs because the inclusion of some magnetic field lines with local maxima in drift shell construction allows L* to be defined at greater values, providing additional PSD data points beyond the minLCDS that are included in the loss calculation. If Shabansky type 2 and 3 particles were also included, the initial PSD profile would extend further in adiabatic space and therefore further increase the amount of loss to the magnetopause. We have not evaluated Shabansky type 2 or 3 electrons in this study due to the assumptions of *K* partitioning required to compute L* for these populations. However, even this partial evaluation of Shabansky particles shows that the treatment of these magnetic field lines with local maxima has a measurable impact on the amount of loss to the magnetopause experienced by a given population. Additionally, radial transport occurs due to drift orbit bifurcation, which can become competitive with radial diffusion of ultrarelativistic particles at high radial distances (Öztürk & Wolf, 2007) and can increase the rates of radial transport by an order of magnitude in the presence of ULF Pc4 and Pc5 magnetic field fluctuations (Ukhorskiy et al., 2014). The radial transport from drift orbit bifurcation can directly lead to permanent loss through magnetopause (Desai et al., 2021; Ukhorskiy et al., 2011). This radial transport means that Shabansky type 2 and 3 particles likely have a major contribution to losses across the magnetopause during dropout events, particularly when we consider that drift orbit bifurcation typically occurs within 1–2 *R*_*E*_ of the magnetopause (Desai et al., 2021). One key difficulty associated with evaluating the effect of classic drift orbit bifurcation on particle transport and subsequent loss is the breakdown of the adiabatic invariants that occurs for Shabansky type 2 and 3 particles. Radial transport is best evaluated in terms of L* (particularly during geomagnetically active conditions and near magnetopause), but we can not compute the change in L* of a population that does not have a defined L*, confounding our ability to compute and quantify radial transport due to drift orbit bifurcation. Further study is needed to understand the contributions of Shabansky type 2 and 3 particles to dropout events and the role of radial transport associated with drift orbit bifurcation to losses to the magnetopause.

## 5 Conclusion

The permanent loss of radiation belt electrons to the magnetopause is an active research area, with a recent focus on the contribution of radial diffusion to these losses. We have evaluated the losses of relativistic electrons to the magnetopause, using the dropout event during the October 2012 geomagnetic storm as a case study. The loss was evaluated with no radial diffusion, radial diffusion assuming a dipolar geomagnetic field, and radial diffusion against a non‐dipolar geomagnetic field. While both evaluated radial diffusion models significantly increased the electron loss when compared to the scenario with no radial diffusion, up to 10% more loss occurred when radial diffusion was modeled over a non‐dipolar geomagnetic field than with a dipolar background field. Non‐dipolarities in the geomagnetic field are more pronounced at high radial distances and geomagnetically active conditions, so particles undergoing loss to the magnetopause during dropout events sample a significantly non‐dipolar field. Therefore, rigorous evaluation of electron loss via magnetopause shadowing during dropouts requires a radial diffusion model that allows for the effects of the non‐dipolar background field.

Additionally, we evaluated the losses with and without Shabansky type 1 electrons included in the initial PSD distribution, finding that they increased losses to the magnetopause by an average of 0.75% for a given radial diffusion model. These results show that the treatment of magnetic field lines with local maxima impacts the calculated loss of relativistic electrons to the magnetopause during geomagnetic storms, although further studies are needed to fully evaluate the effect of this phenomena on the dynamics of outer radiation belt electrons. In particular, Shabansky type 2 and 3 particles, which undergo drift orbit bifurcation, should also be evaluated to determine their contributions to losses across the magnetopause. Radial transport resulting from drift orbit bifurcation should also be studied in the context of sudden dropout events to fully determine the contribution of these particles to loss across the magnetopause during geomagnetic storms.

## Supporting information

Supporting Information S1

## Data Availability

Data from the Van Allen probes and the Global Positioning System (GPS) satellites were used for observations of the dropout event. All RBSP‐ECT data are publicly available at the website https://rbsp-ect.newmexicoconsortium.org/rbsp_ect.php (last accessed June 2022), while the GPS data can be accessed through the website https://www.ngdc.noaa.gov/stp/space-weather/satellite-data/satellite-systems/gps/ (last accessed June 2022). OMNI data and the NASA/GSFC's Space Physics Data Facility's OMNIWeb service were also used in this study, which were accessed through the website https://cdaweb.gsfc.nasa.gov/index.html/ (last accessed June 2022). ICME times were obtained from the Wind catalog (Nieves‐Chinchilla et al., 2018). LANLGeoMag (Henderson et al., 2018) is an open‐source code that was used here to calculate the last closed drift shell, and is available from https://github.com/drsteve/LANLGeoMag, and was partially funded through LDRD program award 20150127ER.

## Appendix A Additional Figures

Figures A1 shows the coverage of the Van Allen probes and the GPS satellites that were active during the event evaluated in this study. Figure A2 shows the magnitude of the radial diffusion coeffiecients from the two models used to evaluate outward radial transport.
