# Supplementary material for: Contributions to Loss Across the Magnetopause During an Electron Dropout Event
Source: J Geophys Res Space Phys. 2022 Oct 8;127(10):e2022JA030751. doi: 10.1029/2022JA030751 (PMC9787648; doi:10.1029/2022JA030751)
Supplement: Supplementary file 1 — Supporting Information S1 [file JGRA-127-e2022JA030751-s001.pdf]

Supporting Information for "Contributions to loss across the magnetopause during an electron dropout event"

H. George[1], G. Reeves[2], G. Cunningham[2], M. Kalliokoski[1], E. Kilpua[1], A. Osmane[1], M. Henderson[2], S. K. Morley[2], S. Hoilijoki[1], M. Palmroth[1,3]

[1]Department of Physics, University of Helsinki, Helsinki, Finland

[2]Intelligence and Space Research Division, Los Alamos National Laboratory, Los Alamos, New Mexico, United States of America

[3]Space and Earth Observation Center, Finnish Meteorological Institute, Helsinki, Finland

Contents of this file

1. Figures S1 to S3

2. Tables S1 to S2

Figure S1 – S3

Figures S1 – S3 supplement the discussion of the contribution of Shabansky type 1 particles to the calculated loss. Figure S1 shows that the last closed drift shell (LCDS) values calculated from LANLGeoMag are near-identical whether or not magnetic field lines with equatorial local maxima less than the magnetic mirror values are included. This is shown for the three K values evaluated in this study. Figure S2 shows the PSD of selected populations at the prestorm time and time of minimum LCDS, for PSD composed of both non-Shabansky and Shabansky type 1 electrons. This is in the same format as Figure 3 in the main text that shows the PSD for solely non-Shabansky populations. Figure S3

shows the total loss to the magnetopause and the percentage of the initial population that is lost, again for initial PSD including Shabansky type 1 electrons, in the same format as in Figure 4 of the main text.

Tables S1 - S2

Tables S1 and S2 provide examples of the losses experienced by selected radiation belt electron populations during the October 2012 dropout event with different radial diffusion models. These populations are representative of the response of relativistic populations. Table S1 gives the total loss experienced by outer radiation belt electron populations, comparing the loss of populations composed solely of non-Shabansky electrons to those made up of both non-Shabansky and Shabansky type 1 electrons. Table S2 shows the same but now showing the percentage of the initial PSD that was lost instead of the total loss. Shabansky 2 and 3 particles are excluded from the PSD used to calculate the losses in Tables S1 and S2. The initial PSD of these populations are obtained from the Fokker-Planck DREAM3D simulation, and this is used in conjunction with the LCDS calculated from the LANLGeoMag LCDS code to evaluate the loss across the magnetopause.

References

Brautigam, D. H., & Albert, J. M. (2000). Radial diffusion analysis of outer radiation belt electrons during the October 9, 1990, magnetic storm. *Journal of Geophysical Research: Space Physics*, *105*(A1), 291-309. Retrieved from `https://agupubs.onlinelibrary.wiley.com/doi/abs/10.1029/1999JA900344` doi: https://doi.org/10.1029/1999JA900344

Cunningham, G. S. (2016). Radial diffusion of radiation belt particles in nondipolar magnetic fields. *Journal of Geophysical Research: Space Physics*, *121*(6), 5149-

5171. Retrieved from https://agupubs.onlinelibrary.wiley.com/doi/abs/10.1002/2015JA021981 doi: https://doi.org/10.1002/2015JA021981

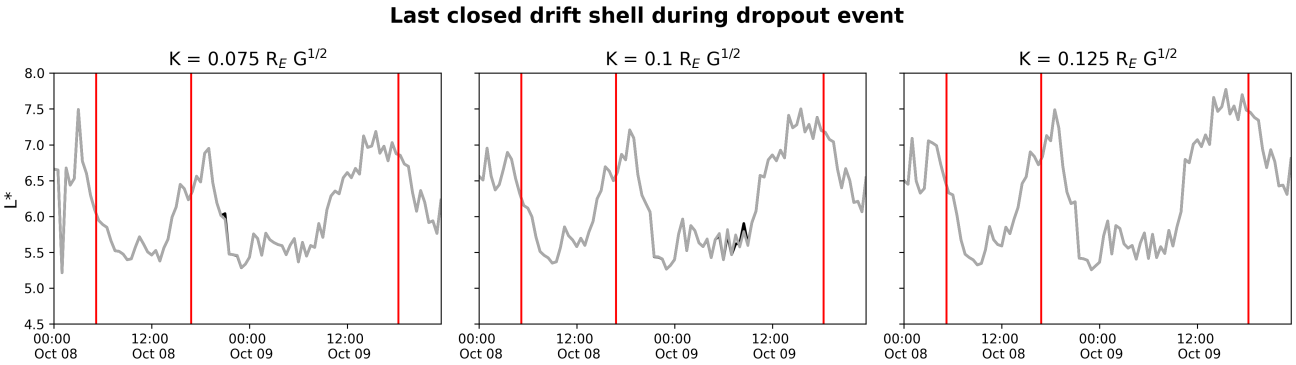

Figure S1. Last closed drift shell from LANLGeoMag for each evaluated K value during the October 2012 dropout event, comparing two types of drift shell construction. Non-Shabansky treatment (black) rejects bifurcated field lines when constructing drift shells, while Shabansky 1 treatment (gray) includes bifurcated magnetic field lines in the drift shells if the local maxima is less than the magnetic mirror value for a given K. These are near-identical due to the methodology and tolerance tests implemented in the LANLGeoMag LCDS code. As a result, the type of drift shell construction does not affect the value of minLCDS used in the loss calculations in this study.

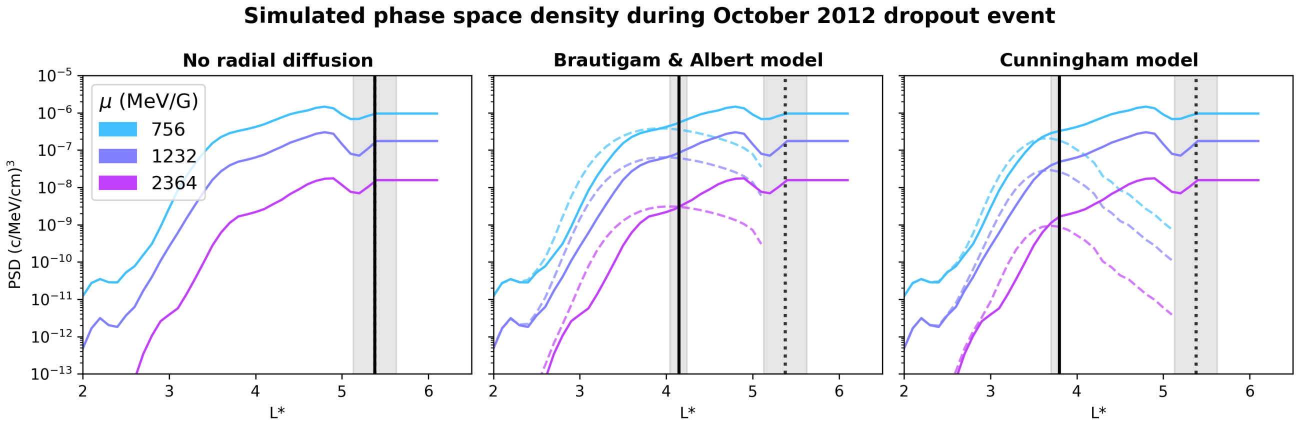

Figure S2. The same as Figure 3 of the main text but now including both non-Shabansky and Shabansky type 1 electron in the PSD profile. Solid lines show the initial PSD and dashed lines show the PSD at the time of minLCDS, for $K = 0.1\ R_E G^{1/2}$ and μ corresponding to the hue of the plot. In each subplot, the dotted vertical line shows minLCDS and the solid vertical line shows L^*_{loss}, the L^* beyond which electrons are lost across the magnetopause. The shaded regions show the uncertainty in minLCDS and L^*_{loss}.

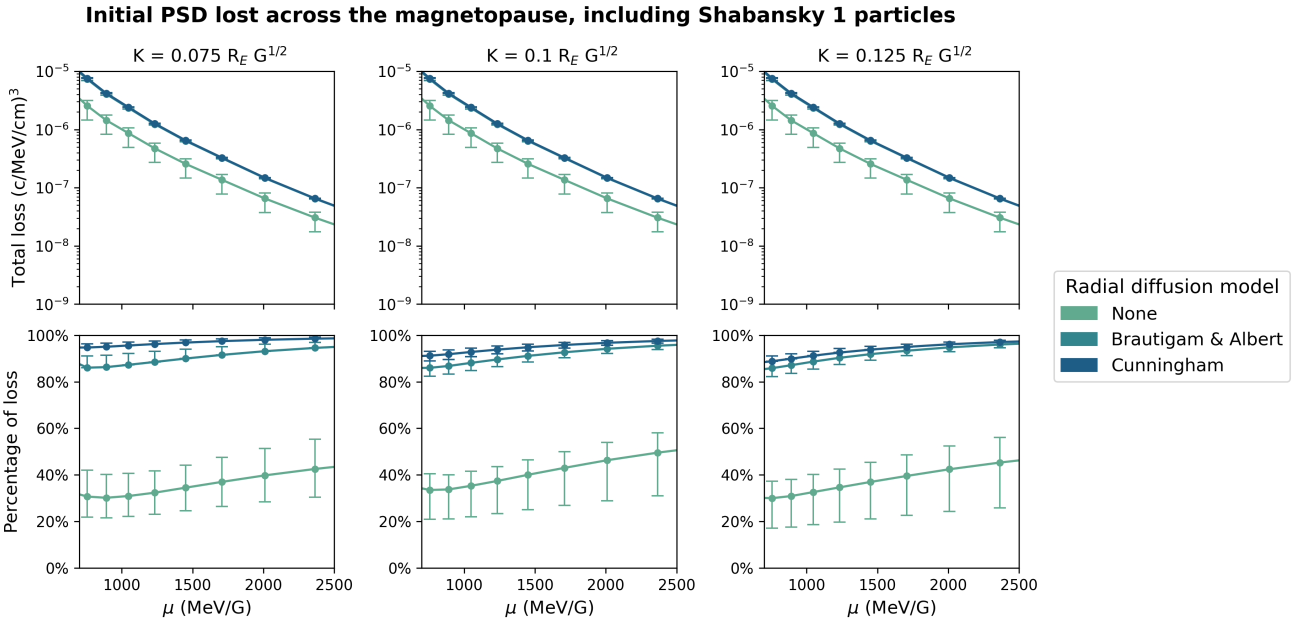

Figure S3. Total (top row) and percentage (bottom row) loss of populations with initial PSD composed of both non-Shabansky and Shabansky type 1 particles, in the same format as Figure 4 of the main text. The columns correspond to different K values and the hue of the plot corresponds to the radial diffusion model. The error bars are calculated from the uncertainty in the minLCDS of ± 0.25, which produces the subsequent uncertainty in both L^*_{loss} and loss in PSD.

Total loss of initial phase space density

K ($R_E G^{1/2}$)	μ (MeV/G)	**No radial diffusion**		**Brautigam & Albert model**		**Cunningham model**	
		Sb 0	Sb 1	Sb 0	Sb 1	Sb 0	Sb 1
0.075	756	5.72×10^{-6}	6.68×10^{-6}	1.78×10^{-5}	1.87×10^{-5}	1.96×10^{-5}	2.06×10^{-5}
	1232	1.05×10^{-6}	1.23×10^{-6}	3.17×10^{-6}	3.35×10^{-6}	3.47×10^{-6}	3.64×10^{-6}
	2364	9.38×10^{-8}	1.09×10^{-7}	2.27×10^{-7}	2.43×10^{-7}	2.38×10^{-7}	2.53×10^{-7}
0.1	756	3.64×10^{-6}	4.86×10^{-6}	1.12×10^{-5}	1.25×10^{-5}	1.20×10^{-5}	1.32×10^{-5}
	1232	6.99×10^{-7}	9.32×10^{-7}	2.00×10^{-6}	2.23×10^{-6}	2.10×10^{-6}	2.34×10^{-6}
	2364	5.63×10^{-8}	7.51×10^{-8}	1.26×10^{-7}	1.45×10^{-7}	1.29×10^{-7}	1.48×10^{-7}
0.125	756	1.82×10^{-6}	2.55×10^{-6}	6.56×10^{-6}	7.29×10^{-6}	6.81×10^{-6}	7.54×10^{-6}
	1232	3.38×10^{7}	4.73×10^{-7}	1.10×10^{-6}	1.23×10^{-6}	1.13×10^{-6}	1.26×10^{-6}
	2364	2.18×10^{-8}	3.05×10^{-8}	5.60×10^{-8}	6.47×10^{-8}	5.67×10^{-8}	6.54×10^{-8}

Table S1. Total amount of the initial PSD that was lost to magnetopause shadowing with no radial diffusion, radial diffusion according to the Brautigam and Albert (2000) model and with the Cunningham (2016) radial diffusion model. This was calculated by summing the initial PSD distribution that was transported beyond the minimum last closed drift shell during the sheath portion of the storm and has units of (c/MeV/cm)^3. The loss is shown for selected electron populations that correspond to relativistic energies with significant bounce motion. Sb0 and Sb1 refer to the treatment of bifurcated magnetic field lines when determining the initial PSD; the Sb0 criteria produces populations that are composed solely of non-Shabansky electrons while the Sb1 criteria additionally includes Shabansky type 1 electrons in the initial PSD.

Percentage of initial population lost to the magnetopause

K ($R_E G^{1/2}$)	μ (MeV/G)	No radial diffusion		Brautigam & Albert model		Cunningham model	
		Sb 0	Sb 1	Sb 0	Sb 1	Sb 0	Sb 1
0.075	756	27.5	30.7	85.4	86.1	94.5	94.7
	1232	29.1	32.4	88.0	88.5	96.0	96.2
	2364	38.9	42.5	94.2	94.5	98.4	98.5
	Average	30.3	38.3	88.4	89.0	96.1	96.3
0.1	756	27.4	33.5	84.7	86.0	90.4	91.2
	1232	31.0	37.4	88.5	89.6	93.1	93.8
	2364	42.4	49.6	94.8	95.5	97.2	97.5
	Average	37.5	44.2	88.7	89.8	93.2	93.9
0.125	756	23.5	30.0	84.5	85.8	87.8	88.8
	1232	27.5	34.7	89.2	90.3	91.7	92.5
	2364	37.2	45.3	95.4	96.0	96.6	97.1
	Average	32.9	40.5	89.2	90.3	91.7	92.5

Table S2. Percentage of the initial PSD that was lost to magnetopause shadowing with no radial diffusion, radial diffusion according to the Brautigam and Albert (2000) model and with the Cunningham (2016) model, in the same format as Table S1. This is shown for both selected μ values and as the mean loss of populations with μ ranging from 700–2500 MeV/G.
